# Students’ Perceptions of Learning Environment in a Medical College in Mangalore, India: A Mixed-Methods Study

**DOI:** 10.7759/cureus.101176

**Published:** 2026-01-09

**Authors:** Shubhankar Adhikari, Poonam Naik, Imaad Mohammed Ismail, Reshma T M, Muskaan Khan, Kanak Suresh

**Affiliations:** 1 Community Medicine, Chirayu Medical College & Hospital, Bhopal, IND; 2 Community Medicine, Yenepoya Medical College, Mangalore, IND

**Keywords:** dreem, educational environment, medical education, mixed-methods, student perceptions

## Abstract

Background: The educational environment significantly influences medical students’ academic success and well-being. Assessing perceptions of this environment is crucial for improving educational quality. This study was designed to evaluate students’ perceptions of their learning environment in a medical college in Mangalore, Karnataka, India.

Materials and Methods: A mixed-methods study was conducted among medical students in 2023. The Dundee Ready Education Environment Measure (DREEM) questionnaire, which assesses domains including Students’ Perception of Learning (SPL), Students’ Perception of Teachers (SPT), Students’ Academic Self-Perception (SASP), Students’ Perception of Atmosphere (SPA), and Students’ Social Self-Perception (SSSP), along with focus group discussions, was used. Data were analyzed using IBM SPSS Statistics for Windows, version 26 (IBM Corp., Armonk, New York, United States), employing t-tests, ANOVA, and thematic analysis (p < 0.05).

Results: The DREEM score was 122.53/200 (61.26%), indicating a positive environment. The SPT domain had the highest scores, with significant differences observed across academic years (p = 0.006), and SSSP was the lowest (55.50%, p=0.006). Batch differences were significant for SPL, SPT, SPA, and SSSP (p<0.050). Thematic analysis revealed concerns about rote memorization, stress, and limited clinical integration, highlighting needs for interactive teaching and support systems.

Conclusion: The educational environment was generally positive, but social support and stress management need improvement. Enhancing interactive teaching and support systems is essential to optimizing the learning experience and preparing students for professional roles.

## Introduction

The educational environment profoundly shapes medical students’ learning outcomes, professional development, and mental well-being, serving as a cornerstone for high-quality medical education globally [1]. A supportive environment fosters motivation, student engagement, and resilience, enabling students to navigate the rigorous demands of medical curricula. Conversely, stressful or unsupportive settings can impair academic performance and increase burnout, a growing concern in medical education worldwide. The Dundee Ready Educational Environment Measure (DREEM), developed by Roff et al., is a validated tool designed to assess students’ perceptions across five domains: learning, teachers, academic self-perception, atmosphere, and social self-perception [2,3]. Its global adoption underscores its reliability in identifying strengths and gaps in educational settings, making it invaluable for continuous quality improvement [4].

In India, medical education faces unique challenges, including large class sizes, resource constraints, and an emphasis on rote learning, which can hinder critical thinking and clinical application. These issues are particularly pronounced in private medical colleges, where infrastructure and faculty support vary widely. Students’ perceptions of their learning environment provide critical insights into these challenges, guiding institutions toward evidence-based reforms. Studies in India using DREEM have revealed mixed perceptions, with strengths in teacher quality but weaknesses in social support and stress management [5-8]. Understanding these perceptions is essential for fostering a student-centered environment that aligns with global benchmarks for medical education.

Despite its importance, few studies have explored students’ perceptions in medical colleges in South India, particularly using mixed-methods approaches to capture both quantitative scores and qualitative insights. The integration of DREEM with focus group discussions offers a holistic view, revealing not only what students perceive but why they hold these views. Such studies are critical in regions like Mangalore, where medical education is rapidly expanding, yet environmental factors remain underexplored. Addressing these gaps can enhance teaching strategies, student well-being, and institutional reputation, aligning with national goals for healthcare workforce development. Thus, this study was designed to assess students’ perceptions of their learning environment at a medical college in South India, using a mixed-methods approach.

## Materials and methods

This was an explanatory mixed-methods study that employed a combination of quantitative cross-sectional assessment with qualitative focus group discussions at a medical college in Mangalore, Karnataka. The study was conducted among the medical students from the first to fourth years in 2023. The study adhered to the Declaration of Helsinki guidelines, ensuring informed consent, confidentiality, and voluntary participation. Approval was obtained from the Yenepoya Ethics Committee - I (approval number: YEC-1/2024/239).

Sample size

The sample size was calculated using the formula: $n = \left(\frac{Z_{1-\alpha/2}\,\sigma}{d}\right)^2$, where Z_₁-α/2_ = 1.96 (5% significance level), σ = 22 (standard deviation (SD) from previous studies [5]), and d = 3 (precision), yielding a required sample size of approximately 207. Accordingly, 208 students were included.

The sampling frame consisted of official class roll lists obtained from the academic office for each academic year. Probability proportional to size sampling was applied to include 52 students from each academic year. Students were assigned unique identification numbers, and simple random sampling was conducted using computer-generated random numbers in Microsoft Excel (RAND function) (Microsoft Corporation, Redmond, Washington, United States).

Data collection tool and technique

Study Instrument

DREEM Questionnaire: The DREEM is a 50-item questionnaire assessing five domains: Students’ Perception of Learning (SPL) (12 items, maximum score 48), Students’ Perception of Teachers (SPT) (11 items, maximum score 44), Students’ Academic Self-Perception (SASP) (eight items, maximum score 32), Students’ Perception of Atmosphere (SPA) (12 items, maximum score 48), and Students’ Social Self-Perception (SSSP) (seven items, maximum score 28) [2]. Students responded on a five-point Likert scale (4 = Strongly Agree, 0 = Strongly Disagree), with nine negative items reverse-scored. The total score ranged from 0 to 200, with higher scores indicating a more positive environment. The DREEM questionnaire has demonstrated high reliability (Cronbach’s alpha=0.93) globally and specifically in India (Cronbach’s alpha=0.91). Given the tool's extensive validation and high internal consistency in similar South Indian settings, it was deemed a highly reliable instrument for assessing the perceptions of the 208 students in this study. The DREEM Questionnaire has been used with permission from Taylor and Francis, the licensed content publisher.

The present study considered a mean score for each item of ≥ 3 as positive perception, a score of ≥ 2.0 to < 3.0 as an area that could be enhanced, and a mean score of < 2.0 as a problematic area. The guidelines suggested by McAleer and Roff in 2001 [4], as presented in Tables 1, 2, were followed for the interpretation of the overall DREEM score.

**Table 1:** Guide for interpretation of overall DREEM scores DREEM: Dundee Ready Educational Environment Measure

Score	Interpretation
0 – 50	Very poor
51 – 100	Plenty of problems
101 – 150	More positive than negative
151 – 200	Excellent

**Table 2:** Guide for Domain Score Interpretation. Students’ Perceptions of Learning (SPL), Students’ Perceptions of Teachers (SPT), Students’ Academic Self-Perceptions (SASP), Students’ Perceptions of Atmosphere (SPA), and Students’ Social Self-Perceptions (SSSP).

Domain	Score
SPL	0 – 12 Very poor
	13 – 24 Teaching is viewed negatively
	25 – 36 A more positive approach
	37 – 48 Teaching highly thought of
SPT	0 – 11 Abysmal
	12 – 22 In need of some retraining
	23 – 33 Moving in the right direction
	34 – 44 Model teachers
SASP	0 – 08 Feeling of total failure
	9 – 16 Many negative aspects
	17 – 24 Feeling more on the positive side
	25 – 32 Confident
SPA	0 – 12 A terrible environment
	13 – 24 There are many issues that need changing
	25 – 36 A more positive atmosphere
	37 – 48 A good feeling overall
SSSP	0 – 07 Miserable
	8 – 14 Not a nice place
	15 – 21 Not too bad
	22 – 28 Very good socially

Focus group discussions

Based on the quantitative findings, four focus group discussions were conducted to explore domains with mean item scores ≤ 2.0. Each group consisted of four students (total = 16 participants), selected purposively from the study population. Discussions were conducted in the demonstration room of the Department of Community Medicine, lasted 45-60 minutes, and were audio-recorded with the consent of the participants. The recordings were transcribed verbatim within 48 hours. Open-ended guiding questions were used, and member checking was performed to ensure accuracy of interpretations, following COREQ (COnsolidated criteria for REporting Qualitative research) guidelines [9].

Statistical analysis

Quantitative data were analyzed using IBM SPSS Statistics for Windows, version 26 (Released 2018; IBM Corp., Armonk, New York, United States). Descriptive statistics were summarized using mean and standard deviation (SD). Normality of data was assessed using the Shapiro-Wilk test. Independent t-tests were used for comparison between two groups, and one-way ANOVA was used for comparison across more than two groups, with appropriate post-hoc analysis where applicable. A p-value < 0.05 was considered statistically significant. Qualitative data from focus group discussions were analyzed using thematic content analysis [10] in accordance with COREQ guidelines.

## Results

Quantitative results

The study included 208 medical students, with 26.0% aged 22 years, 60.1% (n=125) female, and 39.9% (n=83) male. On the basis of religion, the majority of students were Muslim (49.0%, n=102), followed by Hindu students (44.7%, n=93) and Christian students (6.3%, n=13); 86.1% resided in hostels. The overall DREEM score was 122.53 ± 22.53 (median=124), reflecting a positive educational environment (61.26% of maximum score), with 82.21% of students expressing positive perceptions. The instrument demonstrated high internal consistency in this study population, with an overall Cronbach’s α of 0.91.

As shown in Table 3, in the domain-wise DREEM Score, SPT recorded the highest score (29.18 ± 4.98; 66.31%; α =0.84), with 89.42% of respondents reporting positive perceptions. This was followed by SPL, with 30.18 ± 6.61 (62.87%; α =0.82), and SASP with 19.32 ± 5.65 (60.37%; α =0.76). The SSSP domain recorded the lowest score (15.54 ± 4.64; 55.50%; α =0.68).

**Table 3:** Domain-wise DREEM scores and internal consistency (N=208) *mean score was calculated as a percentage of the maximum score possible for that domain DREEM: Dundee Ready Educational Environment Measure; SPL: Students’ Perception of Learning; SPT: Students’ Perception of Teachers; SASP: Students’ Academic Self-Perception; SPA: Students’ Perception of Atmosphere; SSSP: Students’ Social Self-Perception The DREEM Questionnaire has been used with permission from Taylor and Francis, the licensed content publisher.

Domain	No. of items	Maximum score possible	Obtained score, mean±SD	Mean score*	Students with positive perceptions, n (%)	Cronbach’s α (Reliability)
SPL	12	48	30.18±6.61	62.87	173 (83.17)	0.82
SPT	11	44	29.18±4.98	66.31	186 (89.42)	0.84
SASP	8	32	19.32±5.65	60.37	145 (69.71)	0.76
SPA	12	48	28.02±7.76	58.37	139 (66.82)	0.81
SSSP	7	28	15.54±4.64	55.50	128 (61.53)	0.68
DREEM score	50	200	122.53±22.53	61.26	171 (82.21)	0.91

Table 4 presents the item-wise DREEM scores, showing that the highest-rated aspects were teachers’ knowledge (3.31 ± 0.764), communication skills (3.14 ± 0.871), and patience with patients. Although these items were rated relatively well, none exceeded a mean score of 3.5. In contrast, support for student stress (1.79 ± 1.240) emerged as a problematic area.

**Table 4:** Scoring of each item in the DREEM questionnaire DREEM: Dundee Ready Educational Environment Measure

Item No.	Item statement	Mean Score (SD)
Students’ Perception of Learning		
1	I am encouraged to participate during teaching sessions	2.79 (1.012)
7	The teaching is often stimulating	2.56 (0.951)
13	The teaching is student-centred	2.72 (0.817)
16	The teaching is sufficiently concerned to develop my competence	2.62 (1.033)
20	The teaching is well-focused	2.78 (0.890)
22	The teaching is sufficiently concerned to develop my confidence	2.75 (0.962)
24	The teaching time is put to good use	2.48 (1.002)
25	The teaching overemphasizes factual learning	1.25 (0.819)
38	I am clear about the learning objectives of the course	2.73 (0.960)
44	The teaching encourages me to be an active learner	2.58 (0.999)
47	Long-term learning is emphasized over short-term learning	2.58 (1.069)
48	The teaching is too teacher-centred	2.36 (1.129)
Students’ Perception of Teachers		
2	The teachers are knowledgeable	3.31 (0.764)
6	The teachers are patient with patients	3.00 (0.904)
8	The teachers ridicule the students	2.29 (1.233)
9	The teachers are authoritarian	2.12 (1.220)
18	The teachers have good communication skills with patients	3.14 (0.871)
29	The teachers are good at providing feedback to students	2.68 (1.044)
32	The teachers provide constructive criticism	2.59 (0.979)
37	The teachers give clear examples	2.94 (0.826)
39	The teachers get angry in class	2.32 (1.194)
40	The teachers are well prepared for their class	2.92 (0.929)
50	The students irritate the teachers	1.87 (1.271)
Students’ Academic Self-perception		
5	Learning strategies that worked for me before continue to work for me now	2.35 (1.066)
10	I am confident about my passing	2.43 (1.075)
21	I feel I am being well prepared for my profession	2.32 (1.062)
26	Last year’s work has been a good preparation for work	2.36 (1.040)
27	I can memorize all I need	1.80 (1.092)
31	I have learnt a lot about empathy in my profession	2.79 (1.028)
41	My problem-solving skills are being well-developed	2.48 (0.973)
45	Much of what I have to learn seems relevant to a career in medicine	2.79 (0.9167)
Students’ Perception of Atmosphere		
11	The atmosphere is relaxed during the ward teaching	2.18 (1.144)
12	This institute is well scheduled	2.44 (1.097)
17	Cheating is a problem in this institute	2.25 (1.317)
23	The atmosphere is relaxed during the lectures	2.50 (1.059)
30	There are opportunities for me to develop interpersonal skills	2.35 (1.157)
33	I feel comfortable in class socially	2.50 (1.146)
34	The atmosphere is relaxed during seminars/tutorials	2.52 (1.085)
35	I find the experience disappointing	2.69 (1.086)
36	I can concentrate well	2.21 (1.104)
42	The enjoyment outweighs the stress of studying medicine	1.74 (1.312)
43	The atmosphere motivates me as a learner	2.18 (1.134)
49	I feel able to ask the questions I want	2.47 (1.112)
Students’ perception of self-performance		
3	There is a good support system for students who get stressed	1.79 (1.240)
4	I am too tired to enjoy this course	2.43 (1.206)
14	I am rarely bored in this course	1.91 (1.229)
15	I have good friends in this institute	2.81 (1.150)
19	My social life is good	2.17 (1.285)
28	I seldom feel lonely	2.07 (1.315)
46	My accommodation is pleasant	2.34 (1.320)

Tables 5, 6 show the associations of the domains with demographic variables and academic year. Sex and place of residence showed no significant differences across domains (e.g., SPL: P=0.160 for sex, P=0.230 for place of residence; Table 5). However, batch differences were significant for SPT (P=0.006), SPA (P=0.042), and SSSP (P=0.006), with the 2020 batch scoring highest (Table 6).

**Table 5:** Association of domain-wise DREEM scores with sex and place of residence ^a ^p-values derived using Independent t-test; ^#^p-values derived using Mann–Whitney U test DREEM: Dundee Ready Educational Environment Measure; SPL: Students’ Perception of Learning; SPT: Students’ Perception of Teachers; SASP: Students’ Academic Self-Perception; SPA: Students’ Perception of Atmosphere; SSSP: Students’ Social Self-Perception

Grouping Variable	Domain	Mean±SD		p value
		Male	Female	
Sex	SPL	29.39±6.70	30.70±6.54	0.16
	SPT	32.79±5.52	33.41±6.20	0.31
	SASP	19.13±6.08	19.44±5.38	0.70^a^
	SPA	27.80±7.74	28.17±7.81	0.49
	SSSP	15.15±4.75	15.79±4.58	0.32
Place of Residence		Day-scholar	Hostelite	
	SPL	28.69±7.62	30.42±6.43	0.23
	SPT	31.76±7.28	33.40±5.68	0.25
	SASP	18.66±6.51	19.43±5.51	0.50^a^
	SPA	28.21±8.54	27.99±7.66	0.81
	SSSP	16.03±5.62	15.46±4.48	0.60

**Table 6:** Association of domain-wise DREEM scores with the medical school year * P-values derived from ANOVA with post-hoc tests. DREEM: Dundee Ready Educational Environment Measure; SPL: Students’ Perception of Learning; SPT: Students’ Perception of Teachers; SASP: Students’ Academic Self-Perception; SPA: Students’ Perception of Atmosphere; SSSP: Students’ Social Self-Perception

Grouping Variable	DREEM Domain	4^th^ Year, mean±SD	3^rd^ Year, mean±SD	2^nd^ Year, mean±SD	1^st^ Year, mean±SD	p value
Medical School Batch	SPL	28.84±7.89	30.79±6.42	30.65±5.43	30.66±6.29	0.54
	SPT	30.52±6.75	34.31±5.15	34.11±5.31	34.16±5.46	0.01*
	SASP	18.05±6.47	19.48±5.78	19.74±5.06	20.30±4.88	0.13
	SPA	25.64±8.46	28.56±6.61	28.89±6.93	29.48±8.58	0.04*
	SSSP	14.09±4.75	16.85±5.23	16.06±3.77	15.27±4.33	0.01*

Qualitative results

To complement the DREEM inventory findings, focus group discussions were conducted to explore students’ perceptions of the learning environment in depth. Thematic analysis revealed eight key themes, including challenges with memorization, academic stress, and needs for enhanced teaching and support systems, as summarized in Table 7.

**Table 7:** Thematic analysis of student perceptions in the learning * Qualitative data underwent thematic content analysis, generating themes manually per COREQ guidelines [9] COREQ: COnsolidated criteria for REporting Qualitative research

Theme	Subtheme	Codes	Verbatim Quotes
Learning Approaches and Methods	Emphasis on Memorization Over Conceptual Learning	Exam-oriented learning and focus on factual learning: The curriculum heavily emphasizes preparing for exams, leading students to prioritize rote memorization of factual information over deep understanding	"*In our curriculum, there's a lot of focus on memorizing facts for exams. Understanding the concept sometimes takes a back seat.*" (MBBS III-Part 1)
		Repetitive teaching methods and inconsistent teaching quality across subjects: Teaching methods are often repetitive, and the quality of teaching varies significantly across different subjects, creating an uneven learning experience	"*Certain facts have already established and need to be known as they are, so memorisation is very important for us in certain aspects and subjects*." (MBBS III-Part 1)
		Difficulty in understanding concepts and lack of application in clinical settings: Students often struggle to understand complex concepts due to the emphasis on memorization, and there is a noticeable gap between theoretical knowledge and its practical application in clinical settings.	"*In subjects like biochemistry and pharmacology, it's about knowing details precisely for exams*." (MBBS I)
Challenges in Memorization	Information Retention Issues	Time-consuming and extensive subject matter: The process of memorizing the vast amount of subject matter is highly time-consuming and overwhelming.	"*Biochemistry is tough because there are so many pathways and enzymes to remember. It's not just about understanding; you have to memorize everything*." (MBBS III-Part 1)
		Frequent revisions are needed due to subject complexity and unengaging textbooks: Constant revisions are necessary to retain the large volume of complex information, which is often made more difficult by unengaging textbooks.	"*Words which are hard to pronounce for the first time of reading something require greater revisions to understand and remember*." (MBBS III-Part II)
		Insufficient visual aids, lack of detailed explanations, and complex terminology: The lack of visual aids and detailed explanations, combined with complex terminology, further complicates the memorization process.	"*The sheer volume of information is overwhelming sometimes. You have to revise constantly to keep up*." (MBBS II)
Students’ Strategies for Effective Memorization	Mnemonics/Codes	Pictographic memorization and use of flashcards: Students use pictographic memorization and flashcards for quick reviews to remember complex information visually and effectively.	"*I use mnemonic devices a lot. They help me remember long lists of drugs and their side effects*." (MBBS III-Part 1)
		Simplifying terminology and using sticky notes for quick review: Simplifying complex medical terminology and using sticky notes for quick reviews aid in easier memorization.	"*Using flashcards for quick reviews and creating summaries of lecture notes have been effective for me*." (MBBS II)
		Color-coded notes and Pomodoro technique: Color-coded notes help in organizing information better, and the Pomodoro technique is used to improve focus and productivity during study sessions.	"I* make colourful notes with different colour pens and highlighters, and I used to do pomodoro technique*." (MBBS III-Part 2)
		Combined study sessions: Group study sessions enhance learning through mutual quizzing and discussion.	"*Integrating case studies into our curriculum would help connect theory to real-life scenarios, enhancing learning.*" (MBBS III-Part 1)
Suggestions for Better Learning	Integrate Theory with Clinical Practice	Promote observational learning and correlation: Emphasize conceptual and practical learning by integrating observational learning;	"*Implementing quizzes and case-based learning scenarios after each module would keep us engaged and reinforce learning*." (MBBS II)
		Shift from exam-focused to practical scenario-based teaching: Increase bedside teaching, conduct more seminars, use visual aids like diagrams and flow charts, implement quizzes after each topic, and include breaks during theory classes for better focus.	"*The one point which I like to add is that, when we have seminars, SRP (Student research project) or group discussions, there should be small breaks in between for our minds to reset because long intervals of listening to something can bore a person*." (MBBS III-Part 2)
Academic Stress	Stress and Enjoyment in Learning	Exam-related stress: The fear of failing exams creates significant stress among students.	"*The idea of failing in examination is definitely one stress, so when you’re learning, the one thing that is stuck in your head is if you don’t learn properly, I am going to fail.*" (MBBS III-Part 2); - -; -
		Disrupted sleep patterns: The large volume of material to cover leads to disrupted sleep patterns as students struggle to keep up with their studies.	"*Exam-related stress affects sleep and daily routine significantly.*" (MBBS III-Part 1)
		Increased anxiety and self-doubt: The overwhelming amount of content and the pressure to perform well cause increased anxiety and self-doubt among students.	"*The volume of material can be overwhelming, affecting our daily routines and sleep*." (MBBS II)
		Active listening and approachability: Teachers who actively listen to students' questions and concerns and are approachable create a more supportive and effective learning environment.	"*One of my medicine professors had to talk to patients with depression, and the way he communicated with her led to her opening up about her depression, which she was quite reluctant to share*." (MBBS III-Part 2)
Teaching Effectiveness	Effective Teachers’ Communication Skills	Non-judgmental attitude: Teachers with a non-judgmental attitude encourage students to participate more freely without fear of being criticized.	"*Teachers who actively listen to our questions and provide clear, detailed answers make a huge difference in our learning*." (MBBS III-Part 1)
		Interactive sessions: Interactive teaching sessions engage students better and make learning more dynamic and enjoyable.	"*Our anatomy professor uses a lot of visual aids and interactive sessions. It makes learning much more engaging and understandable*." (MBBS II)
Areas for Improvement	Allocate Sufficient Time for Each Patient and Students	Addressing student queries thoroughly and providing supportive guidance: Teachers should take the time to address student queries thoroughly and provide supportive guidance to ensure a clear understanding and effective learning.	"*They should give enough time with each patient so they can rant out all their problems.*" (MBBS III-Part 2)
		Creating a comfortable environment: Teachers should create a comfortable learning environment that encourages student participation and engagement.	"I* wish all teachers were as patient and non-judgmental as a few of them are. It would make asking questions less intimidating*." (MBBS III-Part 1)
		Providing feedback: Constructive feedback from teachers helps students understand their progress and areas for improvement.	"*There are times when teachers rush through lectures without ensuring everyone understands. It can be frustrating, especially with complex topics*." (MBBS II)
		Role of faculty: Faculty members should be approachable to support students effectively.	"*I appreciate when faculty members are approachable and take the time to explain things outside of class. It makes a big difference.*" (MBBS III-Part 1)
Curriculum and University Policies	Support System	Mentorship programs and peer support: Mentorship programs and peer support should be strengthened to provide additional guidance and assistance to students.	"*I think they’ve started with some mentorship programmes; we are allotted teachers. I think that’s a good initiative because you know that there is a teacher who you can go to and there’s someone you can share your academic problems and personal problems, I think that is a nice system.*" (MBBS III-Part 2)
		Counselling services: Counselling services should be more accessible to address the mental health and well-being of students.	*"I think that seniors play a very important role in our MBBS life. During our university years, our clinical postings, they really help us a lot by telling us how to deal with each subject in each department...everything*." (MBBS III-Part 2)

These qualitative findings enrich the quantitative DREEM results, particularly explaining lower scores in domains like SSSP. Themes such as academic stress and limited clinical integration highlight areas for curricular improvement.

## Discussion

The current study aimed to assess students’ perceptions of their learning environment at a medical college in Mangalore using a mixed-methods approach. The overall DREEM score of 122.53/200 indicated a positive environment, with SPT scoring highest (66.31%) and SSSP lowest (55.50%). These findings highlight the strength of faculty quality but underscore the need for enhanced social support systems.

Comparisons with global studies reveal consistencies and discrepancies. A Malaysian study reported a similar DREEM score (130/200), reflecting strong teacher perceptions but weaker social support, aligning with the current study’s SSSP findings [11]. Conversely, a South African study noted a lower score (110/200), suggesting greater environmental challenges [12]. Similarly, a study from the United Kingdom (UK) also shows a low score [13].The low SSSP score in our study contrasts with an Australian study, where peer support was robust [14].

Students' perception of learning

The SPL score in this study was 62.87%, with 83.17% of students expressing positive perceptions. Similar studies in the Middle East and the UK report SPL scores ranging from 58% to 66% [15,16]. These studies emphasize student-centered learning and active participation, consistent with the current study's findings that highlight the need for more interactive and stimulating teaching methods.

Students' perception of teachers

The SPT domain scored the highest in this study at 66.31%, with 89.42% of students holding positive views of their teachers. Conversely, some international DREEM-based studies have reported lower overall scores, suggesting greater challenges within the educational environment. The relatively high SPT score in the present study is consistent with findings from studies conducted in the UK, where faculty communication has been identified as a key strength [16].** **Effective communication skills and constructive feedback from teachers were identified as important factors influencing students’ perceptions in the study by Demirören et al. [17].

Students' academic self-perception

The SASP score was 60.37%, with 69.71% of students feeling positive about their academic performance. This is comparable to scores reported in other studies, including those from Saudi Arabia and Ireland, in which SASP scores ranged from approximately 55% to 62% [18-20]. As reported in the study by Jiffry et al., students often cited a need for better integration of theoretical knowledge with clinical practice, echoing the current study's thematic findings on the challenges of memorization and the application of knowledge [21].

Students' perception of atmosphere

The SPA score was 58.37%, with 66.82% of students reporting a positive atmosphere. Studies from other regions, including Nigeria and Nepal, have reported comparable scores, highlighting common concerns related to stress and pressure in medical education [4,22]. A relaxed and supportive atmosphere is essential for effective learning, as reflected in the qualitative data emphasizing the need for less stressful and more engaging learning environments [23].

Students' social self-perception

The SSSP domain scored the lowest at 55.50%, with 61.53% of students expressing positive perceptions. This is a common trend across various studies, where social support systems are often identified as needing improvement [24,25]. The importance of peer support and mentorship programs is highlighted in both the current study and other literature as crucial components of a supportive educational environment [13,14].

Thematic analysis and implications for medical education

The thematic analysis from this study highlights several critical areas within the medical education environment that warrant attention and reform. A prevalent theme is the emphasis on rote memorization over conceptual understanding, which is consistent with findings from other studies across various educational contexts [26]. This focus often leads to students struggling with the retention of vast amounts of information, as they prioritize exam-oriented learning over developing a deeper understanding of the material [27]. As a result, there is a need for curricular reforms that emphasize critical thinking and problem-solving skills [28]. Incorporating active learning strategies, such as problem-based learning and simulation exercises, can enhance students' ability to apply theoretical knowledge in clinical settings, thus bridging the gap between theory and practice [14,27].

Additionally, the present study reveals challenges related to the educational atmosphere, where stress and pressure are common experiences among medical students; similar concerns regarding stressful educational climates have also been reported in postgraduate medical training using different assessment tools [29]. This aligns with findings from other regions, where a supportive and relaxed learning environment is deemed essential for effective education [30,31]. To address these issues, medical institutions must invest in faculty development programs aimed at enhancing teaching effectiveness and communication skills [32]. Teachers who employ interactive teaching methods and maintain open lines of communication with students contribute significantly to a positive educational experience, while collaborative and interprofessional educational approaches further enhance learning experiences across health professions education [33,34]. Furthermore, strengthening support systems, including mentorship programmes and accessible counselling services, is crucial for promoting student well-being and academic success [16,23]. Studies comparing DREEM findings with qualitative interviews further highlight that students’ perceptions of support and well-being are best understood when quantitative scores are interpreted alongside students’ lived experiences [35]. By fostering a supportive and inclusive environment, institutions can help alleviate stress and improve students' social self-perception [12,36].

Student-centered learning approaches, which encourage self-directed learning and provide opportunities for practical application, are vital for preparing students for their future roles as healthcare professionals [14]. The present study highlights the use of memorization strategies such as mnemonics, photographic memorization, and group study to manage extensive curricular content, which is consistent with literature on student learning approaches and is supported by evidence linking educational environments with professional learning behaviours [27,36]. Encouraging collaborative learning and providing resources for students to explore these strategies can further support their academic endeavors. By addressing the identified challenges and implementing the suggested strategies, medical institutions can enhance the educational experience and better prepare students for their professional roles.

Limitations and strengths

The study’s reliance on a single institution limits generalizability, and self-reported data may introduce bias. However, the mixed-methods design provided a comprehensive understanding, and the large sample size enhanced reliability. The integration of qualitative and quantitative data in this study offers a holistic understanding of the educational environment, providing valuable insights for educators and policymakers.

## Conclusions

The study revealed a positive educational environment with strong teacher perceptions but weaker social self-perception. Qualitative findings highlighted rote memorization and stress as key challenges. To align with global health education benchmarks, medical colleges should prioritize student-centered curricula, integrating clinical practice with theory.

Recommendations include implementing problem-based learning, increasing clinical exposure, and establishing robust counselling services. Regular DREEM assessments can monitor progress, while faculty training in interactive methods and student feedback mechanisms can enhance the learning environment. Strengthening mentorship, counselling, and peer support systems is critical to enhance student well-being and reduce stress, ensuring graduates are well-prepared for healthcare roles. Faculty development programs should focus on interactive teaching and communication skills to foster a supportive environment, contributing to national goals for quality medical education.
